# Rosmarinic Acid Enriched Fraction from* Perilla frutescens* Leaves Strongly Protects Indomethacin-Induced Gastric Ulcer in Rats

**DOI:** 10.1155/2019/9514703

**Published:** 2019-03-04

**Authors:** Napapan Kangwan, Komsak Pintha, Suree Lekawanvijit, Maitree Suttajit

**Affiliations:** ^1^Division of Physiology, School of Medical Sciences, University of Phayao, 56000 Phayao, Thailand; ^2^Division of Biochemistry, School of Medical Sciences, University of Phayao, 56000 Phayao, Thailand; ^3^Department of Pathology, Faculty of Medicine, Chiang Mai University, 50200 Chiang Mai, Thailand

## Abstract

Gastric ulcers are a common problem in upper gastrointestinal tract (GI) disorders. Nonsteroidal anti-inflammatory drugs (NSAIDs) are one of the most aggressive factors leading to inducing gastric ulcers. Natural products with lower toxicity and safety are currently sought as a potential source to minimize the effect of the gastric ulcers.* Perilla frutescens* or Nga-mon (in Thai) leaves are rich in rosmarinic acid (RA), which has antioxidant, anti-inflammatory, and anticancer effects. This study investigates the protective effect of ethanolic extract (EE) and aqueous fraction (AF) from* Perilla frutescens* leaves, which are rich in RA, on indomethacin- (IND-) induced gastric ulcer in a rat model. The EE at the doses of 50 and 500 mg/kg body weight, AF at the doses of 50, 250, and 500 mg/kg body weight, or famotidine (a standard drug) were administered for 14 days prior to ulcer induction. The ulceration was performed by intragastric administration of IND. Gross gastric ulcers and biological and histological parameters were examined. The pretreatment with AF had more significant effects than EE, including reduced ulcer index, decreased gastric secretion volume and decreased acidity, but it had an elevated gastric pH relative to the IND-induced gastric ulcer. In a histopathological study, the EE and AF decreased mucosal ulcer, inflammatory infiltration, and degenerative lining cells. The IND-induced expression of inflammatory mediators was significantly attenuated with EE and AF. The experiment also remarkably showed the preservation of mucus and apoptosis protection of EE and AF on a gastric mucosal ulcer. The findings demonstrated that the EE and AF of perilla leaves were capable of protecting the stomach against gastric ulcers induced by IND through anti-inflammatory and antiapoptotic mechanisms that should be further investigated. It is suggested that* Perilla frutescens* leaf could be a potential alternative source of RA as a therapeutic agent and food supplement for NSAID-induced gastric injuries.

## 1. Introduction

Gastric ulcers, which result from an imbalance between the protective and damaging factors in the stomach, are a common problem in upper gastrointestinal (GI) tract disorders [1, 2]. The major cause of gastric ulcers was considered to be the augmented usage of nonsteroidal anti-inflammatory drugs (NSAIDs) for clinical therapeutics [3, 4]. However, the long-term use of NSAIDs has many adverse effects, including GI toxicity and cardiovascular complications [5, 6]. Indomethacin (IND) is a well-known NSAID, and it produces gastric damage that is related to loss of gastric mucosal integrity, generates oxidative stress, suppresses of mucus production, induces apoptosis, and activates inflammation [7, 8]. Proton-pump inhibitors (PPIs) and H_2_ receptor antagonists have been used for the prevention of NSAID-induced gastric ulcers; however, several adverse effects have been reported with prolonged PPI use [5, 9]. Besides medical treatment, several natural products, especially from medicinal herbs with gastroprotective effects, have been intensively sought and reported [10–16].

*Perilla frutescens*, also known as Nga-mon, is one of the most interesting candidates of plants. It belongs to the family of Lamiaceae and is a traditional medicinal herb of several Asia countries, including Thailand [17–19]. There is evidence that the* Perilla frutescens* leaves have numerous pharmacological actions, including antioxidant [20–22], anti-inflammatory [9, 23–26], antiallergic [27], antimicrobial [28], and anticancer [19, 29, 30]. The extracts of* Perilla frutescens* leaves are also effective in the protection of GI diseases, which is related to their phenolic and flavonoid compounds, including rosmarinic acid (RA), apigenin, and luteolin, which are known for their various biological properties and health benefits [26, 27, 31, 32]. There is evidence that* Perilla frutescens* extract attenuates dextran sulfate sodium- (DSS-) induced colitis through generating anti-inflammatory cytokines, suppressing proinflammatory cytokines, and inactivating both NF-*κ*B and STAT3 pathways [32, 33]. In addition,* Rosmarinus officinalis* L., with a rich source of RA, has protective action in ethanol-induced gastric ulcers in rats through increasing the enzymatic antioxidant defense systems but decreasing the production of nitrates and the inflammatory response [26]. In the previous study, perilla leaf at the dose of 50 and 500 mg/kg BW inhibited ACF formation and progression in DMH-initiated colon carcinogenesis in rats [30]. However, the effect of perilla leaves on the protection of NSAID-induced gastric ulcers has not been yet studied and reported. Therefore, this current study aims to investigate the gastroprotective effect of a RA enriched fraction from* Perilla frutescens* leaves on an IND-induced gastric ulcer in a rat model.

## 2. Materials and Methods

### 2.1. Plant Specimen

Fresh leaves of* Perilla frutescens* as Nga-mon were harvested from major cultivation regions in the province of Nan in Thailand. In our previous study, the leaves were certified (voucher specimen number QSBG-K2) by the Queen Sirikit Botanic Garden Herbarium in Chiang Mai, Thailand [19].

### 2.2. Preparation of Ethanolic Extract (EE)

The dried* Perilla frutescens* leaves were pulverized for extraction. The perilla leaf powder was soaked in 1 L of 70% (v/v) ethanol in a shaker (150 rpm) for 12 h at room temperature. The ethanol extraction was performed twice under the same condition. The EE were then filtered and evaporated using a vacuum rotary evaporator (BUSHI, Switzerland) at 70°C. The concentrated aqueous portion was lyophilized into a powder. The dark amorphous powder was stored at -20°C until use.

### 2.3. Fractionated Extraction Based on Liquid-Liquid Partition

The 20 g of EE was redissolved in a hexane-water (1:1) mixture and liquid-liquid partitioned with hexane (Hex), dichloromethane (DCM), and ethyl acetate (EtOAc) to obtain Hex, DCM, EtOAc, and aqueous fractions (AF), respectively. Each fraction was dried under reduced pressure to obtain powder and then submitted for the bioassays. The active components in each fraction of perilla leaves extract were further analyzed.

### 2.4. Determination of RA

The* Perilla frutescens* leaf compounds were identified using high-performance liquid chromatography (HPLC). The dried leaf samples were powdered by a grind and screened through a 380 *μ*m sieve. Each sample was accurately weighed. The combined solution was then transferred into a 25 mL volumetric flask and made up to volume with 70% methanol and filtered through a syringe filter. The EE and RA standards were loaded onto a C18-EPS Rocket column (53 mm × 7 mm, GRACE). The analysis of RA was performed by HPLC with a selected wavelength 280 nm for UV detection. Elution was achieved at a flow rate of 1.0 mL/min at 25°C.

### 2.5. Animals and Experimental Study

**Figure 1:**
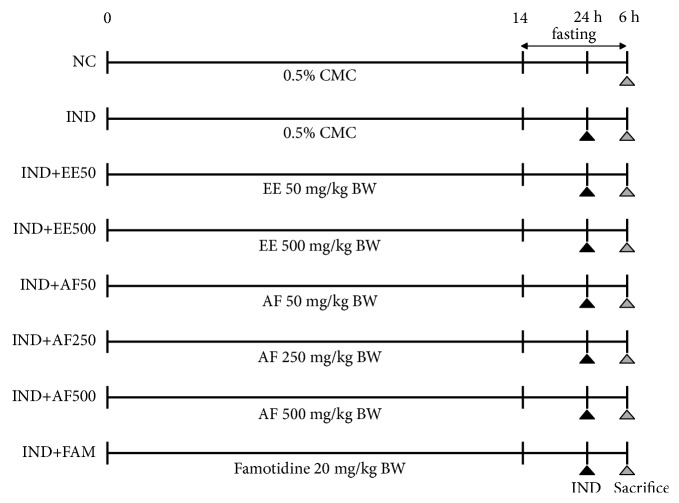
The schematic overview of the experimental design for IND-induced gastric ulcers in a rat model. The pretreatment with EE, AF, or famotidine orally administrated daily for 14 days once per day. Gastric ulceration was performed by administration with IND and sacrificed 6 h later.

Forty-eight male Wistar rats weigh 150–200 g (the National Laboratory Animal Center, Mahidol University, Bangkok, Thailand). All rats were kept in cages that were maintained at 25 ± 1°C in a 12/12 h light/dark cycle for acclimatization. The study protocol was approved by the Institutional Animal Ethics Committee, University of Phayao, Thailand (code number UP-AE5901040009), and all animals were handled in accordance with international guidelines for the care and use of laboratory animals. All rats were divided into eight groups (*n = 6*). The vehicle, EE, AF of* Perilla frutescens* leaves, and famotidine were orally administrated daily for 14 days. All rats were then deprived for 24 h. Gastric ulcers were induced by the administration of IND (40 mg/kg BW) [34] and sacrificed 6 h later. Group 1 was designated as the normal control (NC group) and received only 0.5% carboxymethyl cellulose (CMC). Rats in Group 2 received only IND administration (IND group). Rats in Groups 3 and 4 were pretreated with EE at the doses of 50 and 500 mg/kg BW (IND+EE50 and IND+EE500 group). Rats in Groups 5, 6, and 7 were pretreated with AF at the doses of 50, 250, and 500 mg/kg BW (IND+AF50, IND+AF250, and IND+AF500 group). Rats in Group 8 were pretreated with famotidine at a dose of 20 mg/kg BW (IND+FAM group). Famotidine was used as a positive control as a histamine H_2_ receptor antagonist. All extracts and drugs were dissolved in the 0.5% CMC. The doses of EE and AF used in this study were chosen according to the previous study reported by Khanaree et al. [30]. Gross ulcer and biological and histological parameters were examined after rats were sacrificed. The experimental design is shown in Figure 1.

### 2.6. Collection of the Stomach and Gastric Secretion

The stomach of the rat was collected and gently opened along the greater curvature. Next, the stomach was rinsed and preserved in phosphate-buffered saline (PBS) for macroscopic examination. The gastric contents were centrifuged at 3,000 rpm for 15 min. The supernatant was then collected and used for testing of gastric volume, pH, and acidity.

### 2.7. Gross Ulcer Index and Macroscopic Examination

The gastric mucosa was photographed to examine the ulcer index (UI). The area of gastric ulcer was fixed in 10% formalin for histopathological examination. The UI was scored as described by Sabiu et al. [35]. The percentages of the protective index (PI) of all treatments were calculated by the following of Inas et al. [36].

(1) $$\begin{array}{c} \%\;\;PI=\left[\;\frac{{UI}_{of\;\;IND\;\;group}-{UI}_{of\;\;pretreated\;\;group}}{{UI}_{of\;\;IND\;\;group}}\;\right]\;\;x\;\;100 \end{array}$$

### 2.8. Determination of Gastric Secretion Parameters

The volume of gastric secretion collected was measured and gastric pH was performed with the pH strips. Gastric acidity was determined in the supernatant by titration with NaOH (0.01 N) using phenol red as an indicator. Data were expressed as milliequivalents per liter (mEq/L).

### 2.9. Histological Studies

A small piece of the stomach tissue from each rat was fixed in 10% formalin and embedded in paraffin. The sections were cut and stained with hematoxylin and eosin. The glandular mucosa was analyzed histologically under a light microscope (x400). The histopathological index was blindly reviewed and graded by GI specialists according to criteria that are show in Table 1.

### 2.10. Measurement of Gastric Mucosal Myeloperoxidase (MPO) Activity

The MPO activity was measured using the MPO ELISA Kit according to the manufacturer's instruction (BioVision, Milpitas, CA).

### 2.11. Real-Time Polymerase Chain Reaction (PCR)

RNA was extracted from the gastric tissue using TRIZol® (Invitrogen; Carlsbad, CA). The cDNA was performed with the SensiFAST SYBR® Lo-ROX Kit (Bioline, Singapore) according to the manufacturer's protocol. Real-time PCR amplification was performed using the ABI PRISM® 7000 Sequence Detection System (Applied Biosystems, Foster City, CA). The sequence for the primers for PCR is shown in Table 2.

### 2.12. Measurement of Serum PGE_2_ Level

Plasma was collected and immediately added 10 *μ*g/ml of IND. Then, the plasma was centrifuged at 3,000 rpm for 15 min. Serum PGE_2_ level in the supernatant was measured using the PGE_2_ ELISA Kit according to the manufacturer's instruction. (R&D Systems, Minneapolis, MN)

### 2.13. Determination of Gastric Mucosal Glycoproteins

Sections of the glandular part of the gastric tissue were examined using a commercial periodic acid-Schiff (PAS) staining kit (Ventana Medical System, Tucson, AZ) to visualize mucus production following the manufacturer's instruction. The PAS staining score was expressed as excellent-to-poor preservation regarding mucus production (0-10) [37].

### 2.14. Determination of Apoptosis

Sample sections were stained using the ApopTag Peroxidase In Situ Apoptosis Detection Kit according to the manufacturer's instruction (Millipore, Temecula, CA). The apoptotic index was considered to be the percentage of apoptotic (positive-stained) cells.

### 2.15. Statistical Analysis

The data were expressed as the means ± SEM and assessed by the analysis of variance (ANOVA) test. A Turkey post hoc test was used for the significance of the difference between experimental groups. Differences were considered statistically significant at a* P *value < 0.05.

## 3. Results

### 3.1. Preparation of Ethanolic Extract and Its Fractions of* Perilla frutescens* Leaves

The 1 kg of* Perilla frutescens* leaves was extracted twice with 70% ethanol, and the crude extract yield obtained was 8.61 g. The EE was sequentially liquid-liquid partitioned with solvents including Hex, DCM, EtOAc, and aqua. The AF had the highest yield of 18.27 g, followed by Hex (8.36 g), DCM (5.45 g), and EtOAc (4.10 g) fractions, respectively.

### 3.2. Quantitative Analysis of RA in the EE and Solvent Fractions

**Figure 2:**
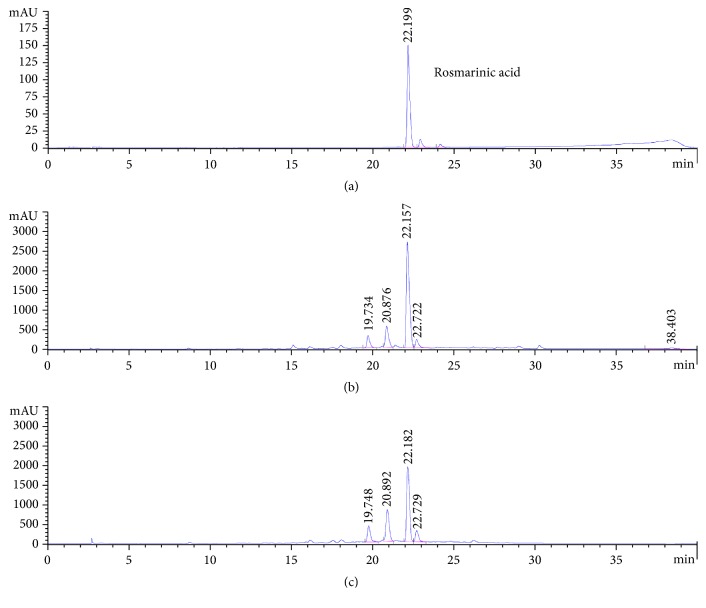
High performance liquid chromatography profile of* Perilla frutescens* leaves at 280 nm. (a) RA standard, (b) EE, and (c) AF.

The HPLC chromatogram revealed the major peak at a retention time (RT) 22.190 min which belonged to RA and a small number of unknown compounds (RT 19.7, 20.8, and 22.7). RA was predominant in EE and AF (431.0 ± 1.12 and 290.1 ± 5.70 mg RA/g) of* Perilla frutescens* leaves. Therefore, EE and AF were chosen for testing the protective effect against IND-induced gastric ulcers in rats. The HPLC chromatogram of RA standard, EE, and AF are presented in Figure 2.

### 3.3. Effect of EE and AF against IND-Induced Gastric Ulcers

**Figure 3:**
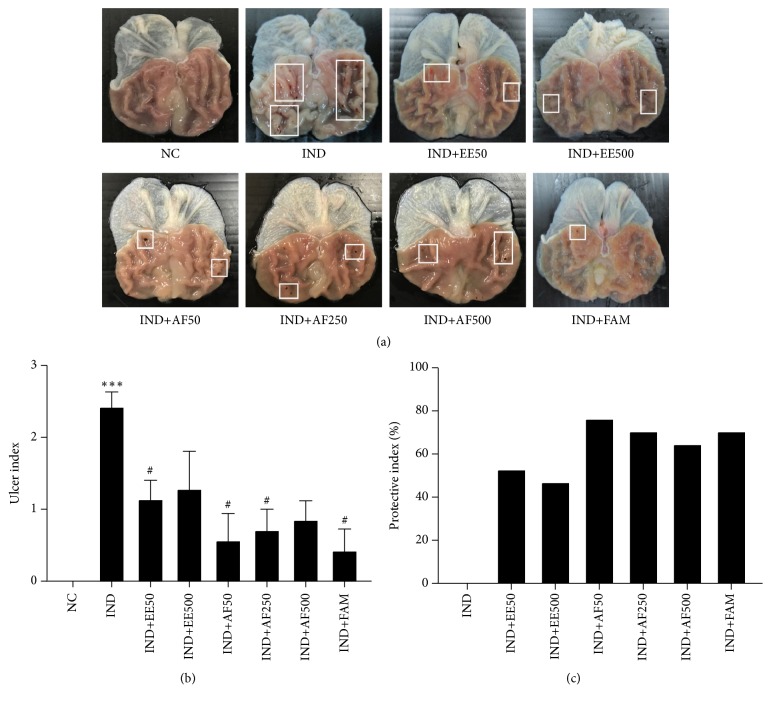
Effect of pretreatment with EE and AF on the gross appearance of the gastric mucosa in IND-induced gastric ulcer in rats. (a) Gross appearance of the stomach. White rectangular indicated the bleeding spots in the ulcer site. (b) Gross ulcer index and (c) The percentage of the protective index. The data are expressed as mean ± SEM (*n=6*). ^∗∗∗^*P* < 0.001* vs*. NC group. ^#^*P* < 0.05; ^##^*P* < 0.01* vs*. IND group.

Rats treated with only IND administration experienced a gastric ulcer in the glandular part of the stomach. The protective effect of EE and AF of* Perilla frutescens* leaves against IND-induced gastric ulcers was demonstrated for the first time. With the pretreatment of EE (50 mg/kg BW) and AF (50 and 250 mg/kg BW), the ulcer index significantly decreased as compared with the IND group (*P* < 0.05) and likewise with famotidine (*P* < 0.05) (Figures 3(a) and 3(b)). The AF of 50 mg/kg BW offered better protection than EE. In addition, the percentages of the PI confirmed that EE and AF of* Perilla frutescens* leaves could protect IND-induced gastric ulcers (Figure 3(c)).

### 3.4. Effect of EE and AF on Gastric Secretion Parameters' Evaluation

**Figure 4:**
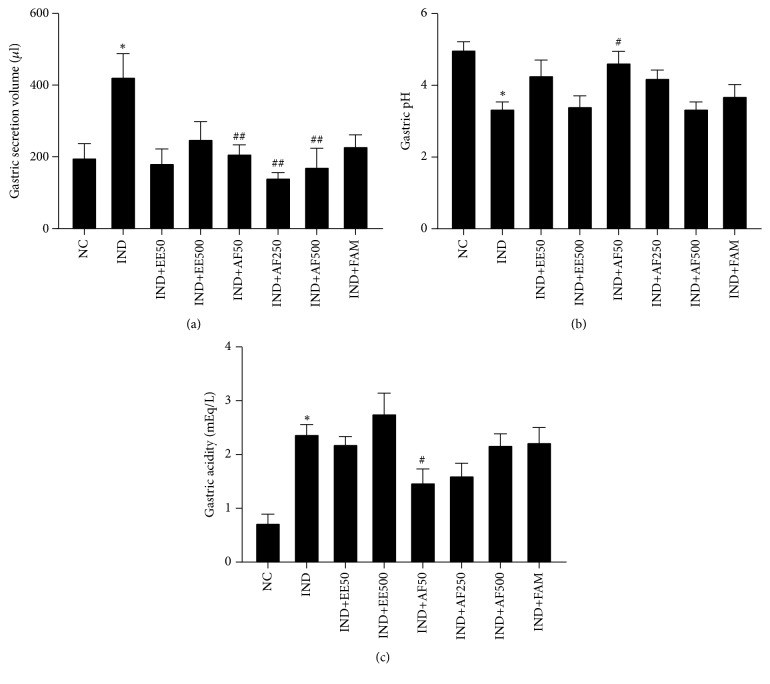
Effect of pretreatment with EE and AF on gastric secretion parameters against IND-induced gastric ulcer in rats. (a) Gastric secretion volume (b) Gastric pH (c) Gastric acidity. The data are expressed as mean ± SEM (*n=6*). ^∗∗^*P* < 0.01* vs.* NC group. ^#^*P* < 0.05; ^##^*P* < 0.01* vs.* IND group.

The gastric secretion parameters, including volume, pH, and acidity, were analyzed to assess the antisecretory effect of EE and AF on IND-induced gastric ulcers. In particular, pretreatment with AF at the dose of 50 mg/kg BW marked a significantly diminished gastric secretion volume (*P* < 0.01) and acidity (*P* < 0.05) when compared with the IND group with a corresponding significant increase in gastric pH (*P* < 0.05) (Figures 4(a)–4(c)).

### 3.5. Effect of EE and AF on Histopathological Examination and Mucosal MPO Activity

**Figure 5:**
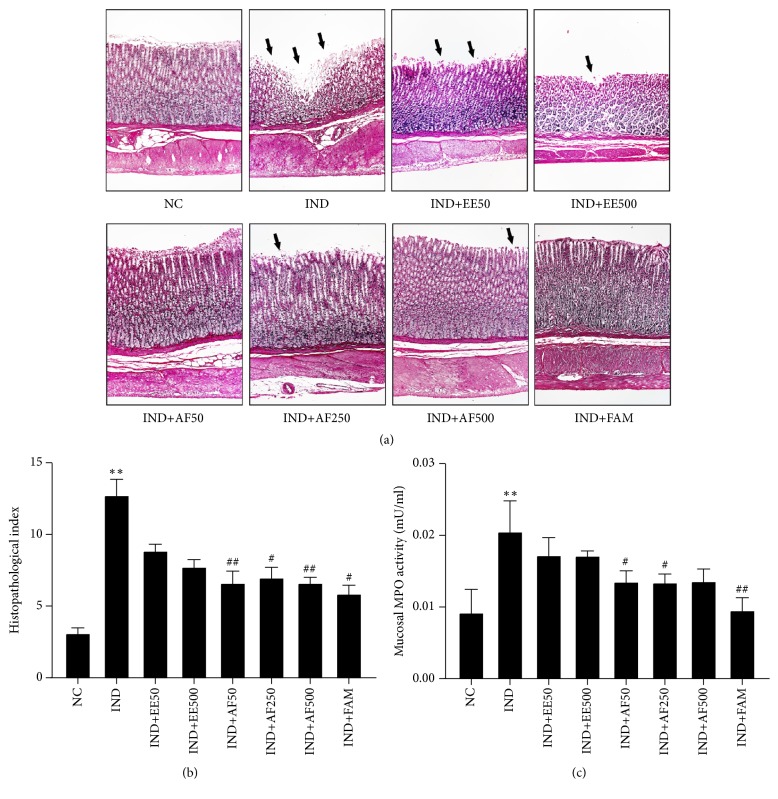
Effect of pretreatment with EE and AF on histopathological observation of gastric mucosa. (a) Light micrograph of the gastric mucosal ulcer by IND (H&E, x100). Extensive disruption to the surface epithelium of gastric mucosa was indicated with a black arrow. (b) The histopathological index was calculated from the intensity of ulceration or erosion in the glandular epithelium, number of petechial, inflammatory cell infiltration, present of polymononuclear (PMN) cell, and expression of degeneration granular lining cell. (c) Mucosal MPO activity. The data are expressed ± SEM (*n = 6*) ^∗∗^*P* < 0.01* vs.* NC group. ^#^*P* < 0.05; ^##^*P* < 0.01* vs. *IND group.

A histological examination of the stomachs excised from normal control rats showed normal layers of the stomach wall. The stomachs of rats subjected to IND administration showed severe disruption in the glandular epithelium with erosion, edema of submucosa, inflammatory cell infiltration, focal hemorrhages, and high expression of degeneration of granular lining cells (*P* < 0.01). However, only the pretreatment with AF at the doses of 50, 250, and 500 mg/kg BW significantly declined the histopathological index (*P* < 0.01,* P* < 0.05, and* P* < 0.01, respectively) and its effect was similar to famotidine (*P* < 0.05), as seen in Figures 5(a) and 5(b). Furthermore, gastric mucosal MPO activity was significantly reduced in the pretreatment with AF at the doses of 50 and 250 mg/kg BW (*P* < 0.05), which was similar to pretreatment with famotidine (*P* < 0.01), as shown in Figure 5(c). The pretreatment with EE decreased the histopathological index and MPO activity but without statistical change.

### 3.6. Effect of EE and AF on Gene Expressions of Proinflammatory Cytokines

**Figure 6:**
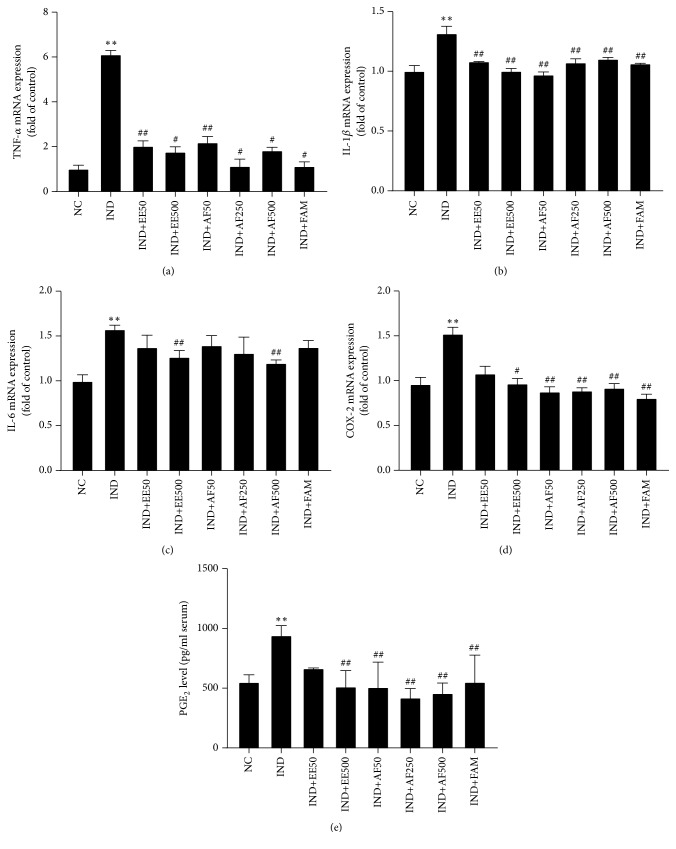
Effect of pretreatment with EE and AF diminished of the expression of proinflammatory cytokines gene, COX-2 gene, and PGE_2_ level on IND-induced gastric ulcer in gastric rats. (a) TNF-*α*, (b) IL-1*β*, (c) IL-6, (d) COX-2 mRNA expressions, and (e) serum level of PGE_2_. The data are expressed as mean ± SEM (*n=6*). ^∗∗^*P* < 0.01* vs.* NC group. ^#^*P* < 0.05; ^##^*P* < 0.01* vs. *IND group.

An IND-induced gastric ulcer is associated with the increase of proinflammatory cytokines. Thus, the mRNA expression of proinflammatory cytokines in gastric tissue after being induced by IND was observed using real time PCR. This study found that the mRNA expressions of TNF-*α*, IL-1*β*, and IL-6 (Figures 6(a)–6(c)) were all significantly elevated after administration of IND compared to the normal control rats (*P* < 0.01); however, the pretreatment with EE and AF significantly suppressed the expressions of TNF-*α* and IL-1*β* (*P* < 0.05), which was similar to pretreatment with famotidine. In addition, the pretreatment with EE and AF at the dose 500 mg/kg BW significantly reduced the IL-6 level (*P* < 0.05 and* P* < 0.01, respectively).

### 3.7. Effect of EE and AF on mRNA Expression of COX-2 and PGE_2_ Level

The mRNA expression of COX-2 was significantly elevated with the administration of IND compared with the normal control rats (*P *< 0.01) but significantly reduced in pretreatment with EE at a dose of 500 mg/kg BW (*P *< 0.05) and all doses of pretreatments with AF (*P *< 0.01) (Figure 6(d)). Furthermore, the production of serum PGE_2_, the major metabolite of COX-2, was measured and was found to be significantly elevated in the IND group compared with the normal control (*P *< 0.01). However, the production of PGE_2_ was remarkably suppressed by pretreatment with EE at the dose of 500 mg/kg BW and all doses of AF (*P *< 0.01) (Figure 6(e)). This result was consistent with COX-2 expression, as shown above.

### 3.8. Effect of EE and AF on PAS Staining

**Figure 7:**
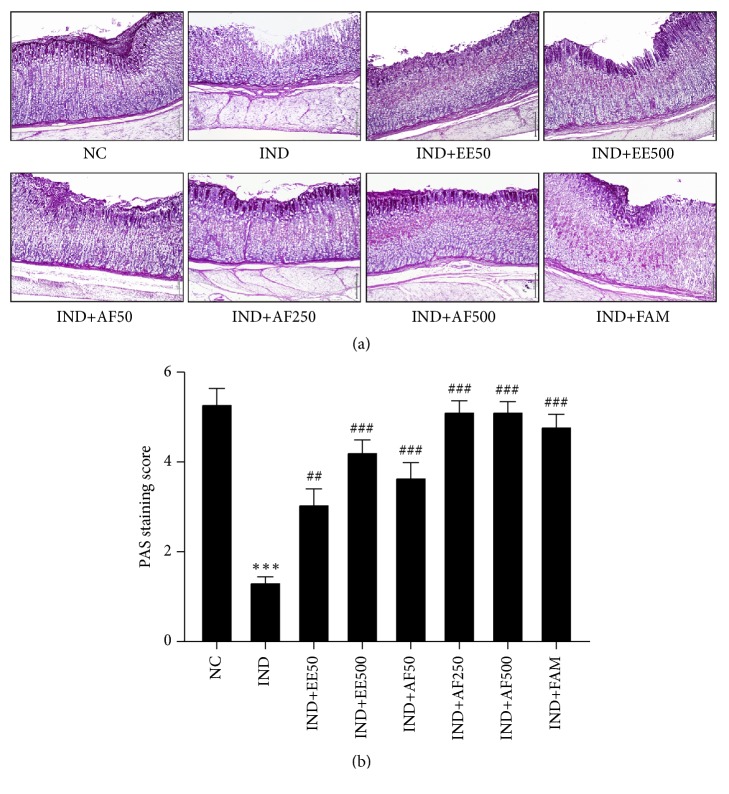
Effect of pretreatment with EE and AF preserved mucus production on gastric mucosa ulcer by IND. The mucus production was detected using PAS staining and score were especially observed in the magenta cells staining on gastric mucosa. The data are expressed as mean ± SEM (*n=6*). ^∗∗^*P* < 0.01* vs.* NC group. ^#^*P* < 0.05; ^##^*P* < 0.01* vs. *IND group.

The presence of mucus in gastric mucosa is important for the protection of gastric ulcers. In the present study, mucus production was examined using PAS staining. The PAS-positive cells were detected especially in the magenta cell staining in gastric mucosa. The PAS-positive cells staining showed a presence of mucus within the goblet cells in the normal control but a loss of mucus in the IND group. However, the pretreatment of EE and AF significantly promoted PAS-positive cells in spite of IND administration (*P *< 0.001), as seen in Figures 7(a)-7(b).

### 3.9. Effect of EE and AF on Apoptosis of Gastric Mucosa

**Figure 8:**
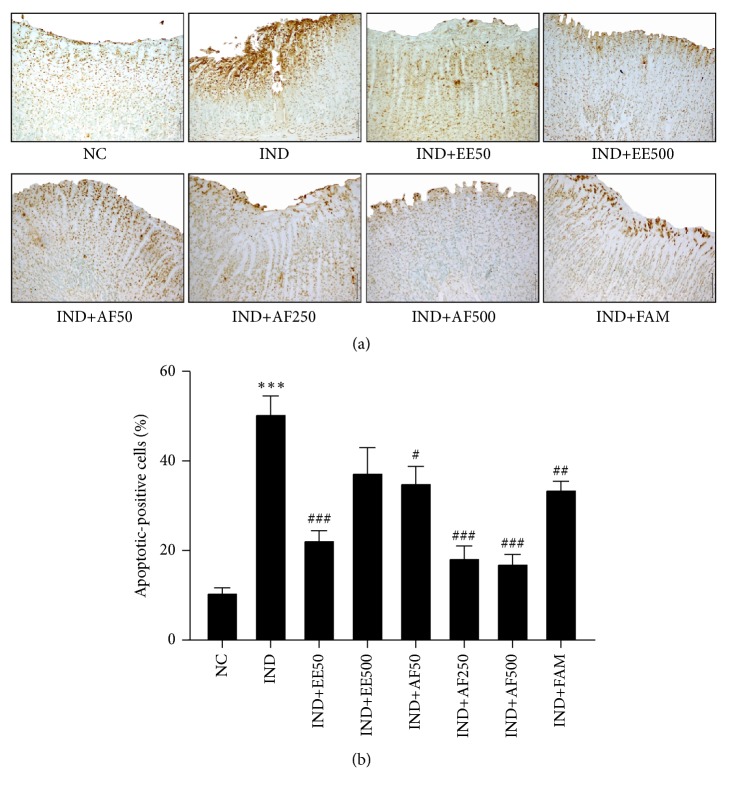
Effect of pretreatment with EE and AF on apoptosis of gastric mucosa. Light micrograph of apoptotic cells (dark brown cells staining) in gastric ulcer induced by IND. The percentage of apoptotic-positive cells was analyzed using light microscopy with high power field (x400) from at least 5 fields. The data are expressed as mean ± SEM (*n=6*). ^∗∗∗^*P* < 0.001* vs.* NC group. ^#^*P* < 0.05; ^##^*P* < 0.01; ^###^*P* < 0.001* vs.* IND group.

This study was conducted to assess the effects of EE and AF against IND-induced apoptosis in gastric mucosal cells. The apoptosis-positive cells were detected in the scattered cells of the ulcer as a dark brown staining. The groups pretreated with EE at the dose of 50 mg/kg BW, AF at the dose of 50 mg/kg BW and higher doses (250 and 500 mg), had significantly fewer apoptotic-positive cells than the IND group (*P *< 0.001, < 0.05, and < 0.001, respectively) (Figures 8(a)-8(b)).

## 4. Discussion

There is evidence that the extracts of* Perilla frutescens* leaves are effective in the protection of GI diseases. The protective effect is related to their antioxidant and anti-inflammatory activities of phenolic and flavonoid compounds, especially, RA [26, 27, 31, 32]. In the present study, our results from the HPLC analysis demonstrated that the RA was predominant in EE (431.0 ± 1.12 mg RA/g) and AF (290.1 ± 5.70 mg RA/g), as shown in Figure 2. Therefore, the EE and AF were chosen to examine the protective effect of RA enriched fraction from* Perilla frutescens* leaves in IND-induced gastric ulcers. In addition, the pretreatment with EE and AF markedly reduced the gastric ulcers induced by IND in the rats. These changes were accompanied by an increase in volume and acidity and a decrease in pH of gastric secretion. The results confirmed that RA enriched from EE and AF of* Perilla frutescens* leaf was responsible for the potential antiulcerogenic and antisecretory activities. Besides RA,* Perilla frutescens* leaf extract also contains other constitutes, including apigenin and luteolin [38, 39]. The antioxidative and anti-inflammatory activity of these compounds have been reported [40]; thus, they could act synergistically with RA in an antiulcerogenic effect. Our finding is the first report to demonstrate the strong gastroprotective effect of* Perilla frutescens* leaf extract on IND-induced gastric ulcers.

It has been shown that the RA acts as a protective agent against gastric ulcers caused by IND via several mechanisms [13, 26], such as the increase of neutrophil infiltration [41]. The neutrophils decrease natural ulcer healing by their lipid peroxidation, yielding free radicals such as O_2_^−^ and H_2_O_2_ [12]. Thus, the suppression of the infiltration of neutrophils by antioxidant and anti-inflammatory activities of RA could be an important recovery mechanism [12, 42]. By histopathological assessment, this result found that the administration of IND in rats induced gastric mucosal damage such as inflammatory infiltration and degenerative granular lining cells. The pretreatment with EE and AF resulted in normal arrangement of mucosal layers and suppression of the neutrophils infiltration, similar to the rats receiving famotidine. This finding was confirmed by determination of MPO activity. The MPO found in neutrophils catalyzes the reaction of Cl^−^ and H_2_O_2_ to form HClO, which is toxic to microorganisms and host tissues [12]. This result demonstrated a significant inhibition effect of pretreatment with AF, but only slightly with EE, on subsequent MPO generation induced by IND administration. The inhibition effect of neutrophil infiltration and MPO might be due to the presence of RA in EE and AF from* Perilla frutescens*.

Ulcerogenic factors, including NSAIDs, Helicobacter pylori, and ethanol, can upregulate proinflammatory cytokines in gastric mucosa [3, 6, 7, 42]. In the current study, the anti-inflammatory properties of both extracts of perilla leaves were determined. This finding showed that the proinflammatory cytokines, including TNF-*α*, IL-1*β*, and IL-6, were elevated after IND administration. In contrast, the pretreatment with EE and AF suppressed the production of these cytokines. These findings were in accordance with Amaral et al. [26] in that* Rosmarinus officinalis *L. with enriched RA had an antiulcerogenic effect on ethanol-induced gastric ulcers in rats through increasing the oxidative markers and antioxidant defense systems while decreasing the production of nitrite or nitrate and the inflammatory response. In addition, Huang et al. [25] demonstrated that the perilla extract downregulated proinflammatory mediators via the inactivation of the NF-*κ*B signaling pathway in LPS-stimulated RAW264.7 cells. Moreover, Tanaka et al. [43] demonstrated that the COX-2 expression was elevated after IND administration. Our findings clarify that the administration of IND results in a marked induction of the expression of COX-2 gene and elevation of production of PGE_2_, the major metabolite of COX-2 in serum, demonstrating its participation in IND-induced gastric ulcers. However, the pretreatment with EE and AF decreased the expression of COX-2 and PGE_2_ levels. Therefore, EE and AF exerted a potent anti-inflammatory activity in gastric mucosa via suppressing neutrophil infiltration and proinflammatory production.

The presence of mucus in gastric mucosa is necessary for gastric mucosal protection against IND-induced gastric ulcers [44, 45]. In addition, apoptosis of mucosa cells is another important pathophysiological pathway activated during the formation of gastric ulcer [46]. The imbalance between gastric epithelial cell proliferation and cell apoptosis results in gastric mucosal injury. IND causes apoptosis by increasing proapoptotic protein expressions [46, 47]. Our experiment clearly showed for the first time that the pretreatment with EE and AF could prevent a decrease in mucus production and that it also attenuated the apoptosis of gastric mucosa. The results signified the potential defensive effect of EE and AF on the gastric mucus layer and demonstrated the essential role of mucus as a protective factor against gastric ulcerative ulcers. Long-term use of NSAIDs is harmful; thus,* Perilla frutescens* leaves could be an effective alternative plant source of RA, which might be useful in the management of inflammatory response and protection of apoptosis in gastric ulcers. Further research on anti-inflammatory effects and antiapoptosis effects of* Perilla frutescens *leaves might lead to the discovery of new pharmacological products in the prevention and clinical treatment of inflammatory diseases in the GI tract and other tissue ulcers.

## 5. Conclusion

This is the first report to provide scientific evidence of the gastroprotective activities of the traditional medicine of RA enriched extracts from* Perilla frutescens* leaves through the mechanism of anti-inflammatory activity, an increase in mucus production, and protection of apoptosis in gastric mucosa. We have shown that EE and AF had a protective effect on IND-induced gastric ulcers by decreasing aggressive factors, including gastric secretion volume and acidity, leading to the reduction of apoptosis in gastric mucosa but an increase in mucus production and protection. The anti-inflammatory activity of perilla extracts was demonstrated by inhibiting the synthesis of prostaglandins, which is the end product of the cyclooxygenase pathway. The perilla extracts also had the ability to suppress the infiltration of neutrophils and proinflammatory cytokines accordingly, decreasing the severity of the ulcer. In summary, this study reports the gastroprotective effect of perilla leaf extracts through their antiulcerogenic, anti-inflammatory, and antiapoptosis properties. Perilla leaves might be an alternative potential source to be further developed for dietary supplements, such as tea or capsules, and therapeutic agents for the prevention of GI tract inflammation induced by alcohol, NSAIDs, bacteria, and viruses.

## Figures and Tables

**Table 1:** Microscopic assessment of histopathological index.

Lesion criteria	Score	Descriptive remarks
(1) Intensity of ulceration or erosion	N x 2	Ulcer, Erosion < 1mm
	N x 3	Ulcer, Erosion > 1mm
	N x 4	Perforation
	1	Ulcer, Erosion depth <1/3
	2	Ulcer, Erosion depth 2/3
	3	Ulcer, Erosion depth 3/3
(2) Number of petechiae	0	0
	1	1-5
	2	5-9
	3	≥10
(3) Inflammation	0	None
(monocytes or neutrophils)	1	Lower 1/3 portion of mucosa
	2	Middle portion of mucosa
	3	Upper 1/3 portion of mucosa
(4) Density of inflammation	0	None
	1	≤ 25 cells/HPF
	2	> 25 - 50 cells/HPF
	3	> 50 cells/HPF
(5) Present of PMN (neutrophils)	0	No
	1	Yes, scatter
	2	Yes, aggregate
(6) Degeneration granular lining cell	0	No
	1	Yes, mild morphology change
	2	Yes, scatter and morphology change
	3	Yes, aggregate and morphology change

*∗*N= number of ulcers each stomach.

**Table 2:** The sequence for the primers for real-time PCR.

Gene	Forward	Reverse
COX-2	5'-GCCCACCAACTTACAATGTGC-3'	5'-CATGGGAGTTGGGCAGTCAT-3'
TNF-*α*	5'-AAATGGGCTCCCTCTCATCAGTCC-3'	5'-TCTGCTTGGTGGTTTGCTACGAC-3'
IL-1*β*	5'-CACCTCTCAAGCAGAGCACAG-3'	5'-GGGTTCCATGGTGAAGTCAAC-3'
IL-6	5'-TCCTACCCCAACTTCAATGCTC-3'	5'-TTGGATGGTCTTGGTCCTTAGCC-3'
GAPDH	5'-GACATGCCGCCTGGAGAAAC-3'	5'-AGCCCAGGATGCCCTTTAGT-3'

## Data Availability

The data and materials supporting the conclusions of this article are included within the article.
